# Investigating Molecular Mechanisms of Embryonic Mammary Gland Development by Bead-Implantation in Embryonic Flank Explant Cultures – A Protocol

**DOI:** 10.1007/s10911-013-9297-1

**Published:** 2013-05-26

**Authors:** Jacqueline M. Veltmaat

**Affiliations:** Institute of Molecular and Cell Biology, A*STAR (Agency for Science, Technology and Research), 61 Biopolis Drive, Singapore, 138673 Singapore

**Keywords:** Embryo, Mammary, Mouse, Bead, Explant culture, Epistasis, Protocol

## Abstract

The involvement of molecular mechanisms in a particular process such as embryonic mammary gland development, can be revealed by modulation of one or several factors that purportedly act in that process. If those factors or their inhibitors are soluble, their function can be tested by loading them onto small inert beads, which are then implanted in cultured explants of the tissue of interest, in this case embryonic flanks. We here describe a protocol for such experiments.

## Introduction

Nowadays, genetic modification is a widely used technique to identify the role of a particular gene in a specific process. But if genetically modified animals are not available, too costly, or don’t survive until the developmental stage of interest; or if the molecule of interest is not encoded by genes (as is the case for e.g. retinoic acid), alternative methods are available to test the potential roles of these molecules. For example, if the molecule of interest is soluble, it can be loaded onto inert beads, which can be applied to, or implanted in, explant cultures of the tissue or organ of interest. Compared to diluting the soluble factor in the culture medium, this method has the advantage of localized application and reduced consumption of the factor.

This technique can be applied to the study of various stages of embryonic mammary gland development. Mammary gland development in mice starts at around embryonic day (E) 10.5, as inferred from the formation of *Wnt10b* positive mammary streaks on the flanks, in the axillae and in the inguinae. On each lateral side of the body, these streaks soon fuse into one continuous line coincident with the asynchronous formation of five mammary disc-shaped placodes, one in the axilla, one in the inguen, and the other three residing on the flank in between forelimb and hindlimb [1]. In the course of 1 to 2 days, these placodes transform into spherical buds which subsequently sprout into the underlying dermis and branch out into the hypodermal fat pad precursor before birth [2].

Klaus Kratochwil developed a culture method in which mammary rudiments (MRs) from E12.5 mouse embryos onwards can undergo normal morphogenesis, albeit it with a delay of about 1 day. For these cultures, Kratochwil dissected individual mammary buds with a few layers of contiguous mesenchyme. He placed these on a filter resting on a metal grid which itself was hanging over a central depression in a special glass culture dish (Grobstein-design), filled with less than 1 ml medium to just touch the filter [3]. This culture method is based on the principle of a Trowell culture, i.e. organ culture at the medium/gas interface on a thin filter membrane supported by a metal grid [4]. For ex vivo culture of MRs at younger stages, including those prior to the onset of mammary gland formation, one can culture a wide band of the flank encompassing all prospective MRs and the limbs [5]. The presence of the limbs prevents retraction of the ectoderm during culture, but has the disadvantage that only MR2, MR3 and MR4 can be monitored, as MR1 and MR5 are covered by the limbs.

This protocol describes the culture of E10.5 and E11.5 flank explants with application or implantation of beads soaked in soluble molecules, to monitor the effect of these molecules on mammary development. In short, beads are loaded with the molecule of interest. Embryos are harvested at ages ranging between E10.5 and E12, and their flanks are dissected for culture as explants. A loaded bead is then grafted underneath the ectoderm [5] or laid on top of it [6]. These explants can be cultured ex vivo for 1–3 days, which is sufficiently long to test the effect of any factor that is loaded onto beads. If culture is extended beyond 3 days, the dermal mesenchyme will stiffen, which interferes with normal growth. For ex vivo experimentation with mammary development from E12.5 onwards, one can use Kratochwil’s culture method [3] or its modification as described elsewhere in this issue [7] and apply beads that are soaked in molecules of interest as described here.

## Protocols

### Preparing Mouse Embryonic Flank Explant Cultures

#### Materials

- Pregnant female mouse. Sacrifice her preferably by cervical dislocation, as CO_2_ may negatively affect tissue viability. It is practical to use a mouse strain that carries a transgenic marker for the mammary line and rudiments, e.g. TOPGAL-F [8] or s-SHIP-GFP [9] for easy analysis of mammary development.
- A (styrofoam) support and needles to pin down and stabilize the sacrificed pregnant female mouse for embryo dissection.
- 70 % EtOH in squirt- or spray bottle, to spray the female’s belly before opening.
- Several sets of sterile dissection instruments (e.g. from Fine Science Tools):
  - Large scissors and blunt serrated forceps to open the mother’s belly skin
  - Smaller scissors and serrated forceps to open the peritoneum
  - Forceps (e.g. Dumont #5) to lift and hold the uterus, and small scissors or Vannas spring scissors to dissect the uterus out of the body
  - 2 watchmaker forceps (e.g. Dumont #5), Vannas spring scissors, 2 Graefe knifes or Tungsten needles, Moria (mini) perforated spoon to transfer embryos
- Sterile DPBS (Dulbecco’s Phosphate buffered Saline with calcium and magnesium, e.g. from Gibco/Invitrogen).
- 100 mm petri dishes.
- 35 mm petri dishes or 6-well culture plates (BD Falcon).
- Stereoscope, preferably set up in a clean room reserved for organ culture experiments.
- Home-made metal support grids, cut from corrosion-resistant stainless steel or aluminium patio screen at 0.7 mm mesh size, in triangles or circles of approximately 30 mm diameter. Bend a 3 mm edge, on which the grids can stand in the dish. Punch holes (e.g. with paper hole-puncher) in the grid for photography of the explants. Alternatively, metal grids without bent edge can be hung over the well of commercially available organ culture dishes (Falcon, BD Biosciences cat# 353037). Wash and sterilize the grids after each experiment by soaking them in 70 % EtOH, drying and autoclaving, and store under sterile conditions. Optionally, metal grids can be replaced by commercially available membrane inserts (Millicell, Millipore cat# PICM03050) for 35 mm dishes/6-well culture plates.
- Nuclepore® Track-Etch membrane PC MB, 13 mm diameter, pore size 0.1 μm (Whatman, cat# 110405). Autoclave the filters (optionally cut in four quarters) in 0.1 % Gelatin (Type A from Acid-cured Tissue, 300 Bloom, Sigma G1890) in H_2_O, at 120 °C (not 140 °C as it will clog the filter pores and lead to tissue death) or bring to a boil on a hot plate. Pick the filters one by one out of the solution while it is still hot and let them dry on the edge (with a minimal surface of contact) of a grid. If the filter is curled when it comes out of the gelatin solution, attach it convex to the grid. Let dry several hours and collect in 20–30 % serum (e.g. Newborn Calf serum) in DPBS. Seal the container and store at 4 °C (possible for several weeks).^1^
- Culture medium: DMEM (Dulbecco’s Modified Eagle Medium) (e.g. Gibco/Invitrogen) to which the following supplements are added^2^ (aliquots of the individual, undiluted supplements can be stored at −20 °C):
  - 10 % Fetal Bovine Serum (e.g. Hyclone cat# SV30160.03)
  - 9 % E9 chick extract (home-made, see [12] for video showing the preparation, but note that they use E11 chick embryos instead of E9) 3
  - 100 U/ml penicillin, 100 μg/ml streptomycin, (e.g. Gibco/Invitrogen cat#1540, 100×)
  - 2 mM Glutamine/GlutaMAX™ (e.g. Gibco/Invitrogen cat# 35050–038, 100×)
- Humidified incubator, set at 5 % CO_2_, 37 °C.
- Wear gloves and wash with 70 % EtOH.

#### Methods

1. Set up a mating pair of mice and check the female daily for presence of a vaginal plug. With normal light (day)-dark (night) cycles, noon of the day a plug is found is considered embryonic day (E) 0.5, gestational day 1.
2. Remove the uterus from female mouse at gestational day 11 or 12 under sterile conditions and transfer to sterile DPBS in 100 mm petri dish. Dissect E10.5 or E11.5 embryos under the stereoscope from the uterus, and remove parietal yolk sac, visceral yolk sac and amnion. Use a spoon to transfer liberated embryos to clean sterile DPBS, taking care not to damage the flanks.^4^
3. Optionally, somites are counted for more precise determination of the developmental stage (instead of chronological age) of the embryo. Under the stereoscope, place the embryo in a small drop of DPBS such that its tail can be placed stably along its forehead. Adjust the light and mirror of the stereoscope until the somite boundaries are visible by transparency. Approximately at the level of the anterior side of the hindlimb is the boundary between somites #24 and #25 in embryos at around E11.5 (Fig. 1). Count from there downwards to the tip of the tail. Note that relative boundaries of limbs and somites shift slightly in the course of development between E10.5 and E11.5, which may create slight errors in counting when comparing litters at E10.5 and E11.5. Nonetheless, this method allows distinguishing the lesser advanced from the more advanced embryos within one litter.

**Fig. 1:**
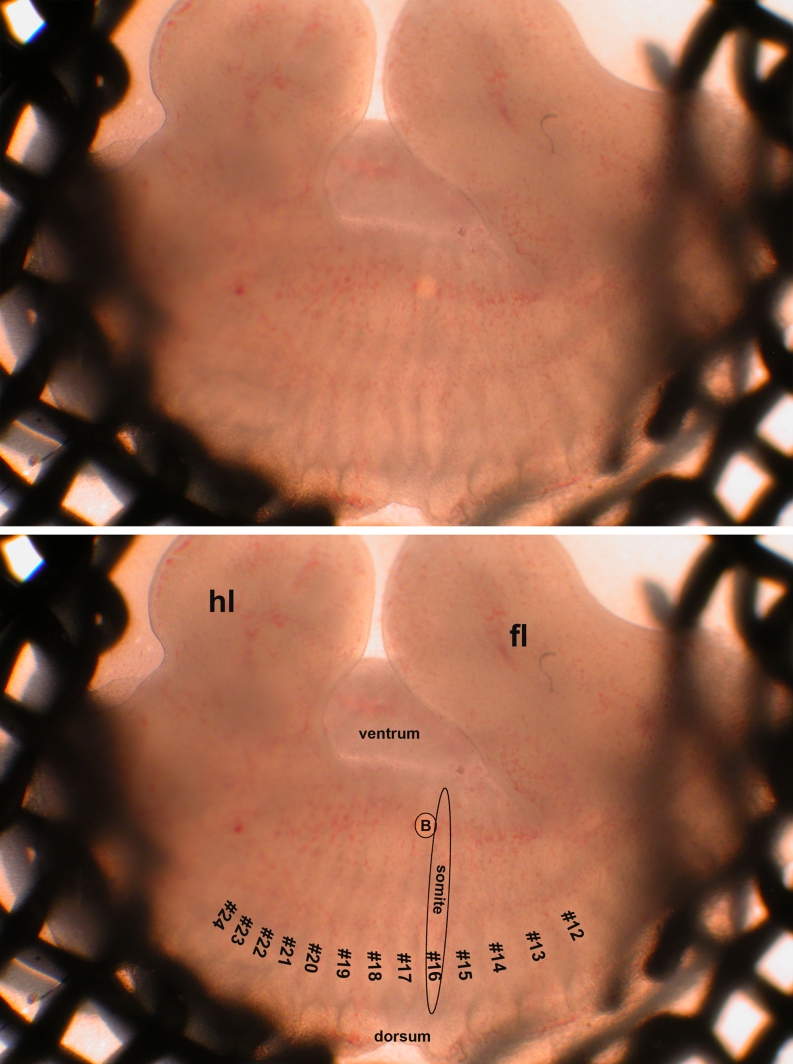
Cultured flank explant with grafted bead. *Lower panel* is an annotated duplicate of the *top panel*. The flank explant rests on a filter that is placed over the hole in a metal support grid. Ellipse indicates one somite, somites #12-#24 are numbered. Circle with B outlines the grafted bead. Other abbreviations: hl: hindlimb; fl: forelimb
4. Deeply submerge one embryo at a time in sterile DPBS. Use Graefe knifes to remove the head and tail. Lay the trunk ventral side down and place the Graefe knifes in and aligning with the dorsal midline of the neural tube, one in the neck, one in the lumbar area. Slide the knives along each other to bisect the embryo along the dorsal midline, through the visceral organs and as near to the ventral midline as possible. Remove all internal organs as they inhibit development of mammary tissue.^5^ The peritoneum should be peeled away, but the dermal mesenchyme and the somites should be retained, as they contain inductive signals for mammogenesis [5]. The heart can serve as a control for culture conditions, and some organs may be useful controls to test activity of the factors loaded onto beads.
5. Set up one of the following three Trowell-type culture dishes according, using forceps:
  - place a bent metal grid into a 35 mm petri dish
  - hang a flat metal grid over the rim of the central well of an organ culture dish
  - hang a Millicell membrane insert in a 35 mm petri dish or 6-well culture plate
6. Using forceps, transfer a washed filter (convex if curled) into the dish with embryonic flanks and push to the bottom of the dish. With closed forceps, gently push a flank onto the center of the filter, mesenchymal side down, ectodermal side facing up. Drag the filter towards the periphery of the dish, and decant the dish so that the explant is no longer submerged. Lift the filter with explant out of the dish and place it on the grid or membrane insert, above the hole and fill the dish with supplemented medium until it just touches the filter. The explant may not be submerged, but has to be situated at the medium/air interface. Avoid air bubbles in the medium and certainly underneath the filter.
7. Place the lid on the dish and the dish in the incubator, and prepare flank explant cultures of the next embryo.
8. In a separate dish, set up a similar Trowell-type culture of a heart, as a positive control for culture conditions. If the heart is not beating at the end of the experiment, culture conditions were not good.
9. In separate dishes, set up similar Trowell-type cultures of an organ that can serve as a positive control for activity of the molecule that is loaded onto beads, e.g. several Fibroblast Growth Factors (most certainly FGF10) will enhance branching morphogenesis of the embryonic lung [14].

### Loading Beads with Factor of Interest

#### Materials

- Pasteur pipettes
- 1 ml individually wrapped Stripettes (e.g. Corning® Costar® available from various vendors)
- 2 or 10 μl pipette
- Microfuge (1.5 ml) and PCR (0.2 ml) reaction tubes, sterile
- DPBS, sterile
- 60 mm petri dish
- Stereoscope, preferably in a clean room reserved for organ culture
- Beads: Heparin-Acrylic beads (Sigma) were the preferred choice for loading e.g. Fibroblast Growth Factors (FGFs) [5] but are no longer available. Affigel beads (BioRad 153–7301 or 153–7301) are a good alternative and can also be used for loading Bone Morphogenetic Proteins (BMPs) or Sonic Hedgehog (SHH). Ion exchange beads (BioRad AG1-XT, 100–200 mesh; 100–150 μm diameter) can be used as well for loading growth factors or their inhibitors [11] and e.g. retinoic acid or its inhibitor citral [6]. When working with acrylic beads, make sure to coat the inside of Pasteur pipettes by aspirating a small volume of serum and washing with DPBS before aspirating beads, so they will not stick to the pipette.
- Bovine Serum Albumin (BSA) (lyophilized, BioReagent; Sigma cat# A9418), as a ‘vehicle’ for growth factors. Prepare a small stock solution of 1 mg/ml in DPBS and store at 4 °C for a few weeks.
- (recombinant) Growth factors (e.g. from R&D Systems) or other molecules of interest. For growth factors: Buy in lyophilized state and reconstitute according to manufacturer’s recommendations and 10× more concentrated than the final concentration in culture, usually in at least 0.1 % (1 mg/ml) BSA. Final concentrations for culture must be retrieved from the literature and by experimentation. Store small aliquots at −80 °C where they can be kept for at least several months. Dilute on ice to final concentration preferably just prior to the experiment, although single use aliquots (e.g. 10 μl) can be made of excess volume at final concentration and stored for at least several months at −80 °C.

#### Methods

1. Using a 1 ml Stripette, transfer a small volume of bead suspension from the commercial stock to a 1.5 ml microfuge tube.
2. Wash these beads several times with DPBS.
3. Using a 1 ml Stripette, transfer beads to a 60 mm petri dish.
4. Under the stereoscope, select beads with a diameter that is about similar to the width of a somite.
5. Transfer sufficient beads for one experiment (add extra for unforeseen loss during experimentation) with a 2 or 10 μl pipette (or mouth pipette with capillary drawn Pasteur pipette) to a sterile 0.2 ml PCR reaction tube. Preferably keep the number (e.g. 40) of beads the same for similar experiments, to minimize loading variation among multiple experiments.
6. Under the stereoscope, aspirate the DPBS entirely with 2 or 10 μl pipette or mouth pipette.
7. Add 10 μl of growth factor/substance e.g. 100 ng/μl recombinant FGF10 in 100 ng/μl BSA in DPBS. Concentration and choice of vehicle may vary depending on the molecule of interest.
8. Suspend an equal number of beads in 10 μl of 100 ng/μl BSA in DPBS (or other vehicle, depending on the molecule of interest) for negative control experiments.
9. Incubate growth factors 1 h at 37 °C with occasional flicking of the tubes to promote equal loading.
10. Store on ice until use. Excess beads in growth factor suspension may be stored up to 1 week at 4 °C.

### Implanting Loaded Beads into the Flank

#### Materials

- 35 mm petri dishes with 3 ml DMEM without any supplements, to wash beads.
- Freshly prepared flank explant cultures, as described above.
- Loaded beads and vehicle/control beads, as described above.
- Sterile fine watchmakers forceps, e.g. Dumont #5.
- Photo-stereoscope, preferably in a clean room reserved for organ culture.
- Humidified incubator, set at 5 % CO_2_, 37 °C.

#### Methods

1. Transfer sufficient test and control beads for one experiment to separate 35 mm dishes with DMEM and place in incubator, 1 h before using them in an experiment.
2. Place the explant culture under the stereoscope, take the lid off.
3. When the experiment is intended to test the role of molecules active in the mesenchyme, use forceps to gently poke a small tunnel just below the surface ectoderm in parallel with the boundaries between the somites. Approach from the dorsal side and move ventrally towards the ventral/hypaxial tip of the somites. Be consistent in placing this tunnel at the same axial level, by counting the somites (#12 is just below the forelimb, this is where MR2 will grow. MR3 grows at the level of somites #15/16, MR4 just above the hindlimb, at the level of somite#24).
4. Pick up a bead with the forceps and gently push in into the tunnel.
5. Alternatively, place a bead on top of the explant at the desired position.
6. Several beads can be placed at separate positions in/on one explant (see e.g. [11]).
7. Perform negative control experiments with vehicle-loaded beads. Alternate the use of left and right flank explants for application of test-beads and vehicle/control beads, because the left and right flank differ in gene activity and mammogenic potential [15].
8. Place test bead against the organ that is chosen as a positive control for the test molecule. Place vehicle/negative control bead in a separate culture of a positive control organ.
9. Place the lids on the dishes and transfer dishes to incubator.
10. Optionally, capture images of all explants at this stage, prior to culture.

### Culture and Analysis

#### Materials

- Optional: Loaded beads and vehicle/control beads, as described above; transfer to DMEM in 35 mm dish and place in incubator, 1 h before using them in an experiment.
- Supplemented medium as above under “Preparing Mouse Embryonic Flank Explant Cultures”; prewarmed at 37 °C.
- 1 ml pipette with sterile tips.
- Sterile fine watchmakers forceps, e.g. Dumont #5.
- Photo-stereoscope, preferably in a clean room reserved for organ culture.
- Humidified incubator, set at 5 % CO_2_, 37 °C.

#### Methods

1. Culture for 1–3 days in the incubator. Replace supplemented medium (as described above, under “Preparing Mouse Embryonic Flank Explant Cultures”) daily with pipette, avoiding air bubbles under the filter. When beads are loaded with growth factors or BSA as vehicle control, they need not be replaced. When beads are loaded with relatively unstable factors (e.g. retinoic acid) and placed on top of the explant, beads may be replaced daily.
2. Capture images of all cultures at the end of the culture period (Fig. 1).
3. Fixate and analyze explants by the method of choice, e.g. visualize the mammary line or MRs by LacZ staining if the embryos carry the TOPGAL transgene as a marker for mammary line and rudiments, or use a fluorescence stereoscope if they carry the s-SHIP-GFP transgene, or perform whole mount in situ hybridization for endogenous *Wnt10b;* or test any other gene of interest.

## Conclusion

The use of beads loaded with molecules of interest in explant flank cultures facilitates the analysis of the role of those molecules in the earliest stages of mammary gland development. This technique can also be used to test epistatic cascades without having to crossbreed different mouse mutant strains with each other.

## Abbreviations

BSA: Bovine Serum Albumin
DPBS: Dulbecco’s Phosphate Buffered Saline
DMEM: Dulbecco’s Modified Eagle Medium
FGF10: Fibroblast Growth Factor 10
MR: Mammary Rudiment

## Footnotes

1 For flank explant cultures of older embryos Voutilainen et al. do not gelatinize the filters, but store them untreated in 70 % ethanol at room temperature and wash in DPBS just before use [7]. Although gelatinization of filters and storage in serum may provide benefits that may be necessary for induction of MRs but not for their later development, it may be worthwhile testing whether gelatinization and serum-coating can be omitted.

2 Note that MR2 and MR4 develop well in all cultured explants, but MR3 failed to develop in the majority of our culture experiments using this medium and metal support grids [5]. However, when we used BGJb medium (Sigma, St. Louis, MO) supplemented with 0.5 % penicillin/streptomycin and 0.2 % ascorbic acid in electroporation experiments as in [10], in combination with Millicell membrane inserts, MR3 always developed. We have not yet tested whether this higher success is due to the medium or the flat support of the membrane inserts as opposed to the quarter Track-Etch filters becoming concave during the culture, as their size is small compared to the hole in the metal grid. This BGJb medium with supplements is also used by [11] for bead implantation experiments. Although the authors of the latter publication do not mention success or failure rates of MR3 formation, this medium may be a good alternative to the one described in the current protocol. Prepare a 10 mg/ml stock solution of ascorbic acid (Cell culture tested, Sigma A4403) in distilled water, filter-sterilize (0.22 μm, Millipore), and store single-use aliquots at −20 °C.

3 Commercially available chick extract (several vendors) may be a practical alternative, but I have not tested these.

4 A movie showing embryo dissection (step 2) is available online at http://www.ijdb.ehu.es/data/11/113424ls/IntJDevBiol-113424ls-SuppMovie1.mp4 [13], and needs to be downloaded (by opting “save link as…”) for viewing. Note that for this protocol, cleaning of surfaces with RNase-away is not necessary, and after dissection, embryos should NOT be collected in RNA-later, but in sterile DPBS.

5 A movie showing flank dissection (step 4) is available online at http://www.ijdb.ehu.es/data/11/113424ls/IntJDevBiol-113424ls-SuppMovie2.mp4 [13], and needs to be downloaded (by opting “save link as …”) for viewing. Note that embryos in the movie were stored in RNA-later and thus look wrinkled/shriveled compared to the fresh embryos one uses for culture experiments. Once the visceral organs have been removed, the subsequent manipulations in this movie should NOT be followed for culture experiments.
